# Determinants of Tobacco Use among Children of a Rural Village in India: An Exploratory Qualitative Study

**DOI:** 10.31557/APJCP.2020.21.1.81

**Published:** 2020

**Authors:** Akanksha Goyal, Ashish Sharma, Sunita Agarwal, Suman Bhansali, Kumar Gaurav Chhabra, Chaya Chhabra

**Affiliations:** 1 *Department of Home Science, University of Rajasthan, *; 2 *Department of Public Health Dentistry, Associate Professor, RR Dental College, Udaipur, *; 3 *Deparment of Preventive and Social Medicine, S.N Medical College, Jodhpur, Rajathan, *; 4 *Department of Public Health Dentistry, Sharad Pawar Dental College, Dmims (Deemed to be University), Wardha, Maharashtra, *; 5 *Department of Pedodontics and Preventive Dentistry, MM College of Dental Sciences and Research, MM (Deemed to be University), Mullana, Haryana, India. *

**Keywords:** Determinants, qualitative research, tobacco, habits

## Abstract

**Background:**

Tobacco is one of the leading causes of preventable deaths. It is both a major social and health problem. According to National Sample Survey Organization of Government of India about 20 million children of ages 10-14 are estimated to be tobacco-addicted. There are grave consequences of tobacco both socially and also on health thus it is of utmost importance to understand the factors leading to its use and to plan strategies to reduce its intake. However, the health implications of this social issue in a rural context have not been explored.

**Aims and Objective:**

this study makes an attempt to explore the health and social implications of tobacco usage by the children below the age of 14 years in hamlet.

**Materials and Methods:**

The present study employed a qualitative study design. Data was collected using focus group discussion and in-depth interview of key informants. Thematic analysis for exploring the explicit and implicit meanings within the data was done. The themes which emerged were knowledge about tobacco and the various products available, children and parents’ tobacco use and habits, the health and social implication of tobacco use, reasons for tobacco use by the children.

**Results:**

It was found tobacco use by the children was very common in the community. Parent, peer pressure, sibling pressure were found to be playing important role in the initiation of tobacco habit by the child. Further illiteracy and lack of awareness was also lead to tobacco use among children.

**Conclusion:**

The study identifies education and awareness of parents about the ill-effects of tobacco play an important role as parents act as role model for their children, thus equal stress should be laid in improving the parental habits. Even raising the prices of tobacco products can help in controlling this habit.

## Introduction

Tobacco is one of the leading causes of deaths which could have been prevented. It is both a major social and health problem. About 4.9 million deaths per annum occurring globally are either directly or indirectly related to Tobacco. These deaths are estimated to rise 10 million by 2030. (World Health Organization., 2017)

Further it has been found out that avoiding tobacco may add 20 years to the life of a teenager. Tobacco usage leads to many health problems, gum disease and tooth loss, chronic lung diseases, like emphysema and bronchitis, lung cancer, oral cancer, coughing spells, wheezing, frequent headaches, increased mucus secretion, reduced physical fitness, poor lung growth and function in those who initiate tobacco usage during their childhood. 

It also leads to worsened health and above all addiction to nicotine which further aggravates the condition and makes leaving the habit of tobacco very difficult. In India alone, nearly 1 in 10 adolescents belonging to the age group of 13-15 years have ever smoked cigarettes and almost half of these reports initiating tobacco use before 10 year of age (Sinha, 2004) 

The tobacco situation is unique in this country because of the availability of vast number of tobacco products both smokeless and smoking. Smoking of cigarette particularly beedis and chewing tobacco (smokeless use) is an age-old practice in India. Further these smokeless products and beedis are very economic as well as easily accessible and available. Thus those belonging to low socioeconomic strata can also easily afford these noxious and intoxicating substances. 

Children and adolescents are the most vulnerable population towards tobacco use and its usage by them is reaching a pandemic level. Patel (1999) and Collby et al., (2000) found that, the addiction seen in the adults for this intoxicating substance is generally initiated during the adolescence. According to the World Bank nearly 82,000-99,000 children and adolescents all over the world begin smoking every day (Jha et al., 2006).

According to National Sample Survey Organization of government of India about 20 million children of ages 10-14 are estimated to be tobacco-addicted. (Patel, 1999; Gupta, 1999) It has also been projected that if the current smoking trends continue nearly 250 million of today’s children will be killed by tobacco (Warren et al., 2000; Heishman, 2001). There are grave consequences of tobacco both socially and also on health thus it is of utmost importance to understand the factors leading to its use and to plan strategies to reduce its intake. 

Studies have been conducted to understand and estimate the prevalence of tobacco usage and its predicting factors. However, the health implications of this social issue in a rural context have not been explored. Thus, this study tries to explore the health and social implications of tobacco usage by the children below the age of 14 in a village near district Mirzapur, Uttar Pradesh. 

*General objective*

To explore the health problems suffered by the children and their family due to tobacco use by the children.

*Aims and objective*

1. To understand the factors leading to tobacco use by the children.

2. To explore the economic impact on the family due to tobacco habit.

3. To understand the health and social implication of tobacco use.

## Materials and Methods

*Methodology*

It is a cross sectional, exploratory study done using qualitative methodology. The study has been conducted in a small Hamlet stretching over a span of 2 km square located in a block Patehre Kala near district Mirzapur, in North east part of Uttar Pradesh during August and September 2017. 

The area was inhabited by, *Adivasi’s* (SC), *Patel’s *(OBC), *Sharma* (OBC), *Vishwakarma* (OBC) and *Gaur *(OBC) caste. 74 percent of the population belong to Scheduled Caste and 26 percent belong to Other Backward Classes. The* Patel’s* were economically sound, affluent and have a higher socioeconomic status than others residing in the hamlet. The population size was 439 out of which 184 are married. There were 98 households in the hamlet. All the people know how to sign however only 68.5 percent of males and 46.9 percent of females have received some sort of education. 

Author (G) has received training in qualitative research methods and she contributed towards development of Focus group discussion (FGD) guide and herself was the Moderator. Author S was the Note keeper had Master’s Degree in Public Health Dentistry. 

Methods of data collection were focus group discussion, key-informant interview using unstructured interviewing . The 2 data method were used to gain in-depth knowledge about the topic of the present study. The focus group discussions conducted by moderator and note-keeper were held with the mothers and fathers residing in the village.

*FGD guide and its Development*

A FGD guide consisting of a series of for guiding questions with probes, each for the Patels, Adivasi fathers and mothers. FGD discussion was developed by the investigators to initiate and conduct the discussion. The guide was reviewed by the experts in the field. 

The first question in the FGD probed over Knowledge about tobacco and the various products available. The second question probed over Children and parents tobacco use and habits. And remaining questions addressed The health and social implication of tobacco use and Reasons for tobacco use by the children. They were conducted to establish a rapport with the community and to gain an understanding about the local, social beliefs and practices related to the tobacco usage and the reasons behind it. 

Three focus group discussions were held. In the first one the members of the discussion were Adivasi mothers. The group constituted of seven females belonging to the age group of 27-30 years. All of them had the same caste and similar socioeconomic status. In the second focus group discussion there were six Adivasi fathers aged 27-30 years who participated, all belonging to similar socioeconomic status. 

The third discussion involved six Patel females belonging to the age group of 23-25 years. Patel’s are among the rich and affluent class present in the Village. 

For key informant interview, three interviews were taken. One was of the community health worker working in the hamlet, the other was of Siksha- Mitra working in the government school located in village near district Mirzapur, in North east part of Uttar Pradesh the third was of a doctor practicing in the bus of a Non-governmental Organization and the fourth was of shopkeeper in village. 

No audio recording device has been used due to the unavailability of the device. The participants for the focus group discussion were selected using convenience sampling. In order to collect information across different economic groups both the Patel’s belonging to higher economic group and Adivasi’s belonging to lower economic cadre were selected. 

In the view of ethical consideration oral informed consent was taken prior to the interview and anonymity of the respondent has been strictly maintained. The data has been kept confidential and was used only for research purpose.

*Universe of study*

A village near district Mirzapur, east Uttar Pradesh.

It is a small village stretching over a span of 1 km 2 located in east Uttar pradesh. It has 98 households in it.

*Study population*

children below the age of 13 residing in Village.

*Respondents*

females, males and children living in the hamlet selected by convenience sampling.

*Data Analysi*s

The notes taken by the note keeper were read twice by the researchers to facilitate accurate interpretation and to ensure that minor details were not missed. Analyst triangulation was also done with moderator and 2 other faculty members who analyzed the comments of the FGD so that data could be interpreted in multiple aspects. Initially the data was coded; which included generating descriptive codes that summarized the quotes. Later, the themes were derived from the codes

## Results

From the focus group discussion, it was revealed that the habit of tobacco use is common among the people living in the community. The habit is not limited to a particular gender or caste nor even to the socioeconomic class. 

The most common form of tobacco used by the females is for cleaning teeth in the form of *Rani-Manjan,* *Gul- Manjan* or *Haathi-chaap manjan*, chewable form of tobacco that is *Surtiis* less commonly used by the females. While in case of males tobacco is used in varied forms which include, chewable form- *Surti, Ghutkacommonly* known here as *Josh*, for cleaning teeth- *Rani-Manjan* or *Gul- Manjan.* Even the males do chew beetle leaf (*Pan*) having tobacco (*Surti*) and slaked lime (*Chuna*) in it. These were the smokeless form of tobacco used by the males while in the smoking form only *Bidi* is smoked by them.

*Knowledge about the various tobacco products*

The focus group provided evidence that the people were not aware about the constitution of the products they were using. Common statement by the *Adivasi’s* was “*in sab cheezo se nasha hota hai*”, which meant all these things have an intoxicating effect. But when asked whether the products that they use does it contain tobacco the answer by* Adivasi* was “*hume naahi pata eeme ka hoot hai*”, we are not aware what these products are made up of.

On the other hand the discussion with the Patel female revealed that they were aware that *Surti, josh* are form of tobacco products and are addictive but were not aware about tobacco being a constituent of *Rani-Manjan , Gul- Manjan or Haathi-chaap manjan* which are sold in the market for cleaning the teeth. 

For the *Adivasi *male tobacco was present in *bidi, surti, paan, josh* but its presence among the teeth cleaning form was questionable. About availability and accessibility of these products, it was found that the tobacco products are easily available and accessible as they are sold at almost all small and big shops present in *Hamlet* and the nearby market *Madihaan.*

A small packet of *Rani-Manjan* costed 3 INR and *Gul- Manjan* was available at 1 Rs, making it economical or cheap. *Surti* does not come in pre-packed packets and people buy it according to their convenience of 3-5 INR each time. Similarly one tobacco pan costs 5 INR. Even children are aware about the availability of the products and the shopkeepers do not hesitate, nor deny selling these products to the children. It was also found out that the shops selling tobacco products can be found near to the schools thus making this product more easily available to the children. 

*Children and parents tobacco use and habits*

Even though being unaware about the presence of tobacco in the various products being use, tobacco useis a common practice in the community. The group discussion gave information about its usage across both the sexes and socioeconomic classes. The children in the community also use the tobacco products as they are easily available and accessible. Children aged 4-5 years can be found using tobacco. Most common tobacco products used by children are, one in the form of cleaning paste, *Rani manjan* or *Gulmanjan*, other forms are *Surti, josh* and even some chew* Pan* which constitutes tobacco, slaked lime and arecanut. Upon probing further it was found that parents are aware about the tobacco habits of their children. When asked why the parents do not take any step towards this, the common reply as, “*hum ka kar sakt hai, humare mana karne ke baad bhi karte hai*”, what can we do, even if tell them not to use they use it. Another female in the group discussion pointed, “*humare packet me se le letehai, ab kaha tak dhyan rakhe*” which clearly showed the children have a habit of taking tobacco from the packet of their parents and parents are being negligent about it. Even parents also provide their children with tobacco products like *Pan, Rani manjan or Gul manjan* when the child asks for it and the parents are themselves using it. Reason for doing this was, “*nahi denge to chori karke le lenge*”. When further asked from where do the children get the money to buy these products? The common answers were, “*hum se lete hai,jooth bol kar*” or “*jab hum apne liye manga te hai tab bacche bhi apne liye le lete hai*”. It was also revealed that children also get tobacco from their friends and most of them eat either due to sibling or peer pressure. One packet lasts for 1-2 days both in case of children and their parents.

*The health and social implication of tobacco use*

The people are unaware about the ill- effects on health of tobacco. When probed about what the warning on the tobacco packet meant there were varied interpretations, for example, “*jyada khane se pait me bhiaisakeeda ho jatahai*” was the interpretation of the sign of scorpion made on the packet of the *manjan. *

The *Patel* females and the *Adivasi* males were aware about that tobacco causes cancer while the group discussion with the *Adivasi* females revealed that they had not heard about cancer, nor were they aware that the products being used by them can cause cancer. 

The knowledge about cancer was poor and the disease was not considered to be serious or life-threatening by the participants. Further almost 10-20 INR per day was spent by the family over the tobacco products, which included both the expenditure by the parents and the child, thus further reducing the amount that could have been saved. This adds to the economic burden of the household but the people have poor understanding about it. Even the participants did not consider tobacco use a bad habit or a habit which hampered their savings. In fact serving tobacco Pan to guests is a common tradition here. 

Several social beliefs have been associated with tobacco use, for example, it helps in defecating and if one does not take it then gas will be formed in the stomach, tobacco paste keeps teeth healthy and tightens the gums. All these beliefs further aggravate the tobacco use. When considering the children, school absenteeism was commonly reported. 

Children generally go to school during the lunch hours to eat the food provided by the government. It was also pointed out because of tobacco use the children have poor diet, “*ab nasha karenge to bhook kaha se lagegi, bus yaha waha khelatra he hai”*

*Reason for tobacco use by the children*

The focus group provided evidence that peer pressure, sibling pressure, tobacco habits of the elders, specially parents play a major role in the initiation of tobacco habits by the child. Even teachers act as a role model in, as the children can see their teachers indulging in this habit thus they also try to relate themselves to it. Further a great amount of exposure of the child to tobacco products occurs when they are asked to buy these products for someone in the family thus making the child aware about the availability, accessibility and affordability of the products. It was also pointed out, *“baccho ko nasha karne me maza aata hai”*.

*Key informant interview*

On interviewing the *Siksha –Mitra* of the government school at *Hamlet* it was found, there is high school absenteeism among the children. They come to school only during the lunch hours to have food provided by the school under the Mid-day meal scheme. 

There is no atmosphere of education at home, nor do the parents pay attention towards the education of their child. Even though the importance of education is being understood by the parents, still no effort is done in this direction. Children can be seen outside school using tobacco products. According to her peer pressure plays an important role in this. She also said, “children can be seen fighting, if the child refuses to share tobacco products with the friends”. These children do not answer in school, do not study at home, “*bus din bhar khelna hota hai”*.

Even the doctor pointed out that tobacco among the children is a problem in the hamlet and he considered parents to be responsible to a large extent for this. As the parents act as role models and children learn seeing them. 

Further lack of education and awareness were also held responsible for being ignorant towards this addictive habit of the child. According to him a lot of effort and time is needed to improve the current tobacco consumption scenario. The shopkeeper runs a small shop in *Hamlet *selling grocery and other consumable goods. He said children do come to buy tobacco from him, and when he denies they give the reason of buying it for their parents. To what extent is this reason true was questionable. Further he adds sometimes the children also bring wheat or rice and buy tobacco in exchange of that. Mostly *Rani manjan *or* Gul manjan *are bought by the children and *Surtiis* not much demanded. The children prefer buying tobacco rather than toffee. 

On interviewing the Community health worker about education of children it was found, inspite of being in fourth or fifth standard children cannot read or write, nor can tell the name of their school. Further it was pointed out that teachers also act as a model in initiation of tobacco habit by the children as they can see their teachers themselves being indulged in this habit. 

Other factors which were pointed out were, “role in the initiation of tobacco by the child are, parents use, sibling and peer pressure, child being asked to buy and bring tobacco products from the shops for the elders at home, leniency at home in providing such products to the child when asked for”. Even media plays an important role in this as, when electricity come the children go to nearby houses having television and generally see the B grade movies which show these bad habits, “*aur bacche bigad jate hai*”.

It was also pointed out that tobacco use is not considered bad in the community, it is normal for the people while alcohol use is considered to be bad and a problem. However, when explained about the ill-effects of tobacco the people do promise leaving the tobacco, still are unable to. Further he adds, “*sablogo ke daant, masude kharaab ho rahe hai par surti nahi chodenge”, “maa baap ko chinta nahi hai baccho ki*”. 

Also he said because children are indulging in tobacco habits they have also started stealing money to buy tobacco or tobacco products itself. As the parents are illiterate they befool their mothers also by selling the rice or wheat present at home and then using that money for buying tobacco products. Even when the parents go out for work they steal the money present in home or sell the onions or potatoes to the local shops and thus buy tobacco. 

Further he added “*Aap dekh sakte hai jab ye bacche nasha karte hai in ki aankhein laal ho jati hai*” that is when they use tobacco their eyes become red. Also, now if they do not use tobacco products on the daily basis as they do, they will not eat the food, be anxious, irritable, speak abusive language, fight with others in order to get tobacco. “*Nashe ki aadat lag gayi hai inhe*”. He also pointed out since the company of these children is not good they also learn abusive language, “*Gaali to inke zibaan par hoti hai*”. It was also found out that the children of Patel because belonging to upper socioeconomic strata do not have such adverse habits and further they are also not allowed to play with the *Adivasi *children thus the role of peer pressure in the initiation of tobacco also diminishes. 

The health worker also accounts presence of illiteracy as a major factor responsible for this habit. As the parents are illiterate they do not understand the harm caused by tobacco nor are they willing to teach the harms to their children. And when the harms are explained to the people using tobacco they revert back saying, “*humare maa –baap ko to kuch nahi hua koi bimari nahi hui tambaku khane se to hume kya hoga*”. Thus the people are not even willing to change neither theirs nor their children habit. However, this is not the case in which the females are literate as they are aware that tobacco is bad thus do not allowtheir children to indulge into this habit. 

## Discussion

Besides various disadvantages of a qualitative study for example the extent of issue cannot be determined, and presence of reporting bias can be there, Qualitative studies can be very useful in evaluation of perceptions and attitudes among study subjects in greater depth and details. It can adapt to the quality of information that is being gathered, and most importantly, qualitative research data is based on human experiences and observations. 

In the past various researches were conducted (Mishra et al., 2014; Basakhetre et al., 2017) on tobacco among children but all of them were conducted quantitatively. In the present study to understand the thinking of child and his or her parents, qualitative study was needed to study in detail the reason behind the consumption of tobacco. 

In the present study it was reported that parents and peer pressure play a huge role in the initiation of the habit by the child. Same results were seen in study conducted by Ullah et al., (2018) in which main reason for consumption of smokeless tobacco among adolescents was consumption of tobacco by their friends. In the study by Chadda and Sengupta (2003) it was reported that Significantly higher proportion of boys smoked if their father or best friends consume tobacco. In a study by Basakhetre et al., (2017)in which tension or stress is the most common reason of consumption of tobacco followed by pear pressure. 

In the present study, people are unaware about the ill- effects on health of tobacco. Contrary results were shown in study by Ullah et al., (2018) and Chadda and Sengupta, (2003) in which majority of study participants had a good knowledge about the harmful effects of tobacco. This may due to lack of education among rural children in the present study.

In the present study, it was reported that several social beliefs have been associated with tobacco use, for example, it helps in normalizing bowel movement (self explanatory and acceptable terms/words) and if one does not take it then leads to decreased bowel movements and Flatulence. Tobacco paste keeps teeth healthy and tightens the gums. Various studies in the past shown same findings that Tobacco is used for several recreational and therapeutic reasons (Charlton, 2004; Goswami et al., 2005; Mishra and Mishra, 2013) and tobacco-based dentifrices are believed to be germicidal aiding in effective teeth cleaning while reducing pain due to dental problems. (Simpson, 1997; Mishra et al., 2014)

In the present study most common form of tobacco used by children was *Rani-Manjan, Gul- Manjan *or *Haathi-chaap manjan, Surti, josh* and even some chew *Pan *which constitutes tobacco, slaked lime and arecanut. While in study by Ullah et al., (2018) conducted in Bangladesh on rural Bangladeshi adolescents, Zarda’ was the most common type of smokeless form of tobacco used. In a study by Mishra et al., (2014) conducted among children in rural area near Bhopal in which Gutkha or pan-masala are the most common form of tobacco used.

In conclusion, the study provides rich information about usage of tobacco by the children of *Hamlet *and the adverse effects of this abhorrent habit. Further it also highlights that parents and peer pressure play a huge role in the initiation of the habit by the child. It can be seen in families where both the parents used some or other form of tobacco children of those families were more likely to indulge in such habit. Also, in-depth interviews revealed that children indulging into this habit were having poor diet, short attendance at school, were unable to read even, resorted to stealing or lying in order to get money to buy tobacco. Further the families of these children in spite having low income were unable to save anything as the saved money was used up in buying tobacco for the parents or the children would take it up to buy tobacco for themselves, thus further deteriorating the condition of the family. Also, the role played by education is being highlighted. In case of *Adivasi’s* as the respondents were uneducated none of them could read the warning present on the packet. Even the sign for cancer printed on the packet of tobacco was interpreted differently by them. Most of the females had never heard the name cancer and were also unaware about the presence of any intoxicating and addictive substance in the powder which they use for cleaning their teeth. While Patel’s being a bit educated as compared to *Adivasi’s* were aware about the harmful effects of tobacco and none of their children indulged into the habit of tobacco use.

*Limitations of the study*

Presence of reporter’s bias as some of the families were aware about that doing *Rani* or *Gul manjan* is bad for health, thus might have led to under-reporting of the problem. As the extent of the problem cannot be quantified further studies should be directed towards estimating the prevalence and also quantifying the factors which play dominant role in provoking the children towards tobacco use.

*Recommendation and Policy implications*

The study clearly shows the need for efforts to be directed in making more stringent laws for curbing tobacco. Also, a close monitoring of whether these laws are being followed or not needs to be done. It also projects importance of education and awareness in curbing the practice. The role played by illiteracy in aggravating the problem is being highlighted very well. Thus education and awareness of parents about the ill-effects of tobacco play an important role as parents act as role model for their children, even equal stress should be laid in improving the parental habits. Even raising the prices of tobacco products can help in controlling this habit. Mass media interventions of long duration using brief, recurring messages to inform and to motivate tobacco product users to quit are needed. 
